# Airway Challenges in Goldenhar Syndrome and Implications for Pediatric Care: A Systematic Review

**DOI:** 10.7759/cureus.105687

**Published:** 2026-03-23

**Authors:** Salma Alkhatib, Noor Chughtai, Maximus S Reese, Carson Maris, Christopher Ahmad, Jared Nichols

**Affiliations:** 1 Medicine, Kansas City University, Joplin, USA; 2 Medicine, Kansas City University, Kansas City, USA; 3 Osteopathic Manipulative Medicine, Kansas City University, Joplin, USA

**Keywords:** difficult airway management, goldenhar, goldenhar syndrome surgery, oav, oculo-auriculo-vertebral syndrome

## Abstract

Goldenhar syndrome, also known as the oculo-auriculo-vertebral (OAV) spectrum, is a rare congenital disorder characterized by craniofacial and vertebral anomalies due to abnormal development of the first and second branchial arches. These structural abnormalities often necessitate frequent surgical interventions in patients with Goldenhar syndrome and may also impair airway visualization, thereby complicating effective tracheal intubation. Despite recognition of the airway risks associated with Goldenhar syndrome, evidence guiding anesthetic and airway management remains limited and unexplored. Current literature consists predominantly of isolated case reports, with each portraying a unique and patient-specific airway scenario. A systematic review of the literature was conducted to evaluate reported anesthetic and airway management strategies in patients with Goldenhar syndrome. A review of literature on Goldenhar syndrome was completed on January 11, 2026, using “PubMed” and “Embase,” and included articles published in the past 10 years. Search terms included “Goldenhar syndrome”, “oculo-auriculo-vertebral syndrome”, “hemifacial macrosomia”, “anesthesia”, “airway”, and “intubation”. Articles were eligible for inclusion if they reported patients with confirmed Goldenhar syndrome undergoing any surgical or procedural intervention with detailed airway and anesthetic management. Exclusion criteria for this review included non-English reports without translation and papers that featured patients without perioperative or anesthetic discussion. The articles were screened to ensure the inclusion criteria were met, and any discrepancies were assessed by the researchers utilizing the Joanna Briggs Institute critical appraisal guidelines. A total of 28 cases met the inclusion criteria, with the majority of patients being infants and children. Cardiovascular and oromaxillofacial surgeries were the most commonly reported procedures. Sevoflurane was the most frequently used anesthetic agent. The most successful first-attempt intubation strategy was found to be Macintosh-style curved blade video laryngoscope followed by Miller-style straight blade video laryngoscope. Successful first-attempt intubation was achieved in only half the cases, while the remaining patients required multiple attempts or escalation to alternative airway strategies. Cases that used the curved Macintosh video laryngoscope and did not yield successful intubation on the first attempt ultimately necessitated invasive rescue interventions such as retrograde intubation and tracheostomy. While several cases that attempted straight Miller blade video laryngoscopy were unsuccessful on the first attempt at intubation, cases using this technique saw eventual success after repeated attempts. Patients requiring multiple attempts or rescue techniques more frequently experienced perioperative complications, including delayed extubation, hemodynamic instability, and one perioperative death. Although definitive airway control was ultimately achieved in most patients, these findings emphasize the potential severity of airway-related complications in patients with Goldenhar syndrome and reinstate the importance of minimizing repeated intubation attempts when possible. Clinicians should be prepared for difficult airway scenarios to minimize airway-related morbidity and mortality in affected children.

## Introduction and background

Goldenhar syndrome, also referred to as the oculo-auriculo-vertebral (OAV) spectrum, is a rare congenital condition arising from the abnormal development of the first and second branchial arches. This disorder of embryogenesis results in a wide array of craniofacial abnormalities, most frequently hemifacial macrosomia, mandibular and maxillary hypoplasia, auricular malformations like microtia, ocular findings such as epibulbar dermoids, and vertebral defects [1]. The clinical manifestation of the disease is highly variable, with severity ranging from unilateral facial asymmetry to severe vertebral malformations. Patients with Goldenhar syndrome undergo surgical procedures frequently due to the structural anomalies that impede the function, appearance, and development of various body systems [1,2]. Craniofacial reconstruction is the most common need for surgery in these patients, as the craniofacial abnormalities cause asymmetry, which can affect chewing, speech, and airway patency. Additionally, this disruption of the normal development of the branchial arches and adjacent neural crest cells contributes to associated cardiac, renal, or neurological abnormalities, all of which increase the need for operative management.

The frequency of operative procedures required makes airway management a critical anesthetic concern in this population and represents one of the most significant challenges in patients with Goldenhar syndrome. Structural abnormalities such as micrognathia, mandibular hypoplasia, and restricted mouth opening can limit visualization and prevent effective intubation [3]. Additionally, cervical spine anomalies such as fused vertebrae restrict proper head positioning, increasing the difficulty during intubation. These features together predispose patients with Goldenhar syndrome to a substantially increased risk for difficult or failed intubation as well as higher perioperative complications [3,4]. Advances in airway management techniques have expanded the range of options for managing patients with difficult airways, such as those with Goldenhar syndrome. Video laryngoscopes, fiberoptic intubations, and supraglottic airway devices like laryngeal mask airways (LMAs) have been developed as alternatives to conventional direct laryngoscopy [5].

Despite longstanding recognition of the airway risks associated with Goldenhar syndrome, the current evidence guiding anesthetic and airway management remains limited and unexplored. Current literature consists predominantly of isolated case reports, with each portraying a unique and patient-specific airway scenario. Although these reports offer valuable insight, there is a lack of consolidated evidence synthesizing the most common airway management approaches, success rates, or complications in this population of patients. This gap in the literature may contribute to uncertainty in perioperative planning and variability in anesthetic practice, particularly for clinicians with infrequent exposure to these patients.

The objective of this review is to systematically evaluate the existing literature describing anesthetic and airway management in patients with confirmed Goldenhar syndrome. A structured analysis of current publications can assist in identifying commonly employed airway devices and techniques, patterns of success and failure, and reported complications. Such an analysis can also highlight other gaps in the literature and areas where further research is required to provide optimal airway management algorithms in these patients. Through consolidating the existing evidence, this review seeks to provide clinicians with a comprehensive overview of current practices and considerations relevant to airway management in this high-risk pediatric population.

## Review

Methods

Search Strategy

A systematic search of the existing literature on Goldenhar syndrome was completed by authors S.A. and N.C. on January 11, 2026, and included case reports published in the past 10 years using the “PubMed” and “Embase” databases. Search terms included “Goldenhar syndrome”, “oculo-auriculo-vertebral syndrome”, “hemifacial macrosomia”, “surgery”, “anesthesia”, “airway”, and “intubation”.

Article Selection and Data Extraction

Articles were eligible for inclusion if they reported patients with confirmed Goldenhar syndrome undergoing any surgical or procedural intervention with detailed airway and anesthetic management. Exclusion criteria for this review included non-English reports without translation and papers that featured patients without perioperative or anesthetic discussion. In the “PubMed” database, 15 results were identified in the original search, with three case reports being excluded for not having enough clinically relevant information. For the “Embase” database, 145 results were first observed. A total of 141 were excluded for not having confirmed Goldenhar syndrome (n = 89), not having surgical or procedural intervention (n = 34), being a duplicate article from the “PubMed” search (n = 7), or not having enough clinically relevant information (n = 11).

Bias Assessment

**Figure 1:**
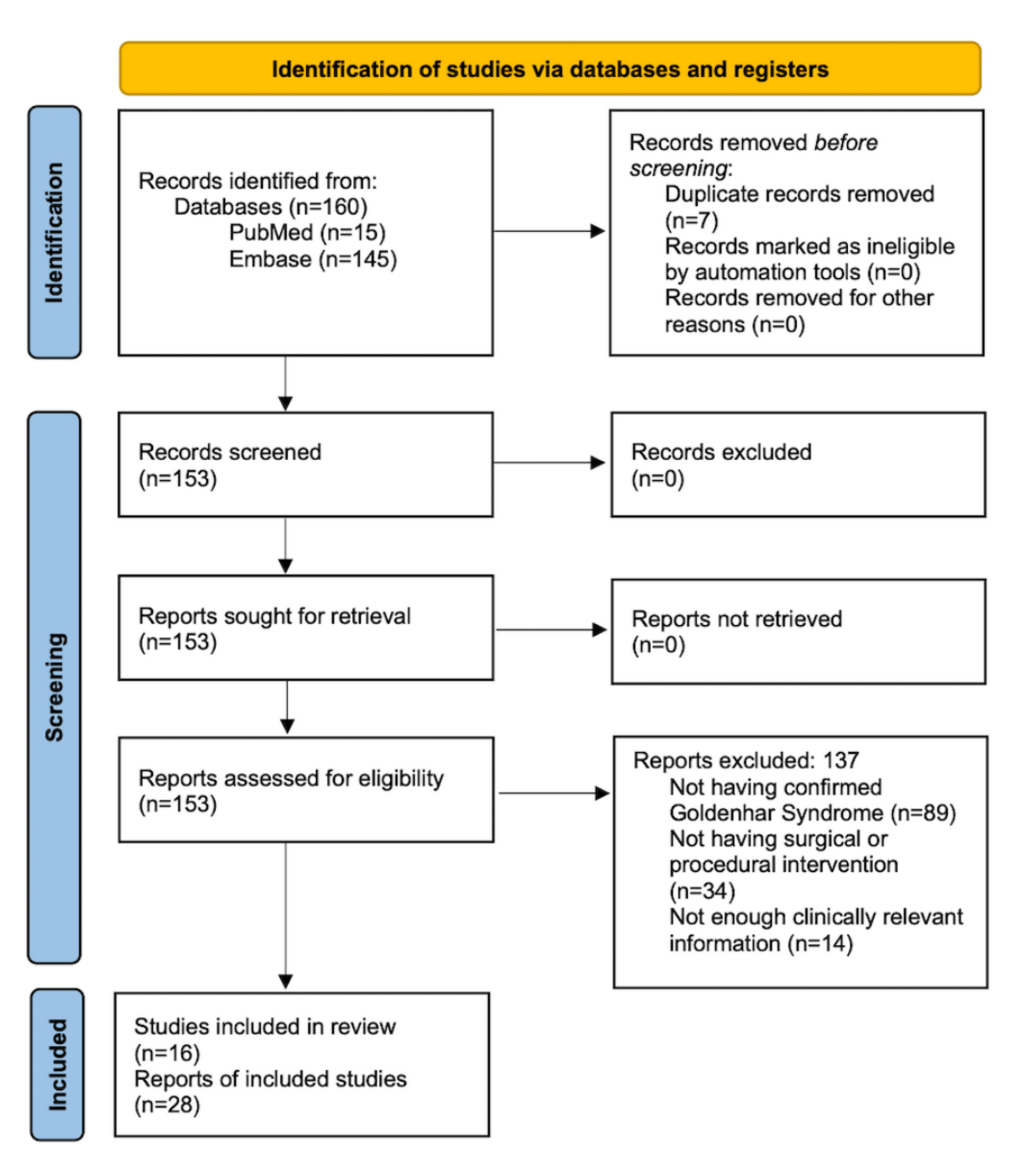
PRISMA flow chart for the article selection process. PRISMA: Preferred Reporting Items for Systematic Reviews and Meta-Analyses

A Preferred Reporting Items for Systematic Reviews and Meta-Analyses (PRISMA) flow diagram is shown in Figure 1, depicting the process for selection [6]. The authors previously mentioned screened the articles briefly to ensure the inclusion criteria were met, and any discrepancies related to article inclusion or exclusion were assessed by the authors utilizing the Joanna Briggs Institute (JBI) critical appraisal guidelines [7]. Potential bias and limitations of this review are considered in the interpretation of the findings.

Twenty-eight confirmed cases of patients with Goldenhar syndrome were found to meet all inclusion criteria and were subsequently included in the final analysis. Study quality was independently assessed using the Newcastle-Ottawa Scale (NOS) for observational studies, evaluating selection, comparability, and outcome domains [8]. Each of the domains was considered, and scores were assigned to each article, with the study being deemed as high quality, moderate quality, or low quality based on scoring greater than 7 points, 5 to 6 points, or less than 4 points, respectively. These findings can be seen in Table 1. Due to the small number of cases, the synthesis is qualitative and descriptive rather than statistical.

**Table 1:** Quality assessment using the Newcastle-Ottawa Scale. Low quality: 0-4; moderate quality: 5-6; high quality: 7-9

Author, year	Newcastle-Ottawa Scale
Choudhury and Kapoor, 2017 [9]	Low
Xing et al., 2023 [10]	Moderate
Singh et al., 2022 [11]	Low
Khan et al., 2017 [12]	Low
Sun et al., 2017 [13]	Moderate
Torres Salazar et al., 2025 [14]	Low
Vitković and Milić, 2016 [15]	Low
Kim et al., 2020 [16]	Low
Molins et al., 2016 [17]	Low
Shukeri et al., 2016 [18]	Moderate
Ishio et al., 2016 [19]	Moderate
Lokhande et al., 2016 [20]	Low
Munasinghe et al., 2021 [21]	Moderate
Lee and Kim, 2021 [22]	Moderate
Eklund and Voronov, 2017 [23]	Moderate
Shankar et al., 2022 [24]	Moderate

Results

Patient Characteristics

The cases were then placed into the unstratified table in order to review patient characteristics and intubation strategies as seen in Table 2 [9-24]. The patients ranged from one month to 42 years, with the majority of the population consisting of infants and children. Of these cases, 13 (46.6%) were male, 12 (42.9%) were female, and three (10.7%) did not report the sex. The surgical procedure planned was analyzed, with cardiovascular surgeries being the most common in 13 cases (46.6%), followed by oromaxillofacial procedures in eight cases (28.6%). Additional procedures included ophthalmologic, orthopedic, and spinal, and neurological interventions reflect the broad range of surgical interventions required in Goldenhar syndrome patients. Several anesthetic agents were used among the patients, with sevoflurane being the most frequent in 17 cases (60.7%), particularly in pediatric patients [9,11-15,17,21]. Propofol was found to be used in five cases (17.9%), mainly among older children and young adults [10,16,19,20,22]. Ketamine and isoflurane were used less frequently, reported in two patients (7.1%) [9,24] and one patient (3.6%) [9], respectively.

**Table 2:** Unstratified airway intubation techniques.

Article (author, year)	Age	Sex	Surgical procedure planned	Type of anesthesia	Airway technique	Intubation difficulty	Successful airway strategy
Choudhury and Kapoor, 2017 [9]	3 months	Female	Ventricular septal defect closure	Isoflurane	Straight laryngoscope and endotracheal tube	None	Straight laryngoscope and endotracheal tube
Choudhury and Kapoor, 2017 [9]	1 month	Male	Pulmonary artery banding	Sevoflurane	Curved laryngoscope and endotracheal tube	None	Curved laryngoscope and endotracheal tube
Choudhury and Kapoor, 2017 [9]	2.5 months	Male	Blalock‐Taussig shunt	Sevoflurane	Curved laryngoscope and endotracheal tube	None	Curved laryngoscope and endotracheal tube
Choudhury and Kapoor, 2017 [9]	8 months	Male	Ventricular septal defect closure	Sevoflurane	Curved laryngoscope and endotracheal tube, straight laryngoscope and endotracheal tube, tracheostomy	Failed intubation	Tracheostomy
Choudhury and Kapoor, 2017 [9]	11 months	Male	Pulmonary artery banding	Ketamine	Curved laryngoscope and endotracheal tube	None	Curved laryngoscope and endotracheal tube
Choudhury and Kapoor, 2017 [9]	10 months	Female	Ventricular septal defect closure	Sevoflurane	Curved laryngoscope and endotracheal tube	None	Curved laryngoscope and endotracheal tube
Choudhury and Kapoor, 2017 [9]	12 months	Male	Ventricular septal defect closure	Ketamine	Curved laryngoscope and endotracheal tube	None	Curved laryngoscope and endotracheal tube
Choudhury and Kapoor, 2017 [9]	12 months	Male	Intracardiac repair	Sevoflurane	Straight laryngoscope and endotracheal tube	5th attempt	None
Choudhury and Kapoor, 2017 [9]	4 months	Male	Atrioventricular septal defect repair	Sevoflurane	Curved laryngoscope and endotracheal tube, straight laryngoscope and endotracheal tube, tracheostomy	Failed intubation	Tracheostomy
Choudhury and Kapoor, 2017 [9]	5.5 months	Female	Blalock‐Taussig shunt	Sevoflurane	Curved laryngoscope and endotracheal tube	None	Curved laryngoscope and endotracheal tube
Choudhury and Kapoor, 2017 [9]	8.5 months	Female	Atrial septal defect closure	Sevoflurane	Curved laryngoscope and endotracheal tube	Failed attempts	Retrograde intubation
Choudhury and Kapoor, 2017 [9]	14.5 months	Female	Correction Tetralogy of Fallot	Sevoflurane	Straight laryngoscope and endotracheal tube	2nd attempt	Straight laryngoscope and endotracheal tube
Choudhury and Kapoor, 2017 [9]	8 months	Male	Blalock‐Taussig shunt	Sevoflurane	Straight laryngoscope and endotracheal tube	3rd attempt	Straight laryngoscope and endotracheal tube
Xing et al., 2023 [10]	18 years	Female	Mandibular distractor	Propofol	Right-angle laryngoscope and endotracheal tube, fiberoptic bronchoscope and cuffed endotracheal tube	3rd attempt	None; procedure done under local
Singh et al., 2022 [11]	8 months	Male	Cataract surgery	Sevoflurane	I-gel supraglottic airway device	None	I-gel supraglottic airway device
Khan et al., 2017 [12]	8 years	Male	Magnetic resonance imaging	Sevoflurane	Laryngeal mask airway	None	Laryngeal mask airway
Sun et al., 2017 [13]	5 years	Female	Cochlear implant surgery	Sevoflurane	Curved video laryngoscope, transnasal fiberoptic bronchoscope with endotracheal tube, transoral fiberoptic bronchoscope	4th attempt	Transoral fiberoptic bronchoscope
Torres Salazar et al., 2025 [14]	21 months	Not reported	Mandible reconstruction	Sevoflurane	Straight video laryngoscope and cuffed endotracheal tube	None	Straight video laryngoscope and cuffed endotracheal tube
Vitković and Milić, 2016 [15]	2.5 years	Male	Palatoplasty	Sevoflurane	Curved video laryngoscope and endotracheal tube	None	Curved video laryngoscope and endotracheal tube
Kim et al., 2020 [16]	18 years	Male	Jaw surgery	Propofol	Transnasal fiberoptic bronchoscope with endotracheal tube	None	Transnasal fiberoptic bronchoscope with endotracheal tube
Molins et al., 2016 [17]	6 years	Not reported	Mandibular hypoplasia correction	Sevoflurane	Hyperangulated video laryngoscope with cuffed endotracheal tube	None	Hyperangulated video laryngoscope with cuffed endotracheal tube
Shukeri et al., 2016 [18]	3 years	Female	Palatoplasty and coloboma repair	Not reported	Straight video laryngoscope, curved video laryngoscope, straight video laryngoscope with endotracheal tube	4th attempt	Straight video laryngoscope with endotracheal tube
Ishio et al., 2016 [19]	12 years	Female	Spinal fusion	Propofol	Curved video laryngoscope with endotracheal tube, curved video laryngoscope with spiral endotracheal tube	3rd attempt	Curved video laryngoscope with spiral endotracheal tube
Lokhande et al., 2016 [20]	42 years	Female	Cesarian section	Propofol	Fiberoptic bronchoscope with cuffed endotracheal tube	None	Fiberoptic bronchoscope with cuffed endotracheal tube
Munasinghe et al., 2021 [21]	5 months	Not reported	Congenital talipes equinovarus correction	Sevoflurane	Straight laryngoscope with endotracheal tube, laryngeal mask airway	3rd attempt	Laryngeal mask airway
Lee and Kim, 2021 [22]	20 years	Female	Mandible distraction osteogenesis	Propofol	Video laryngoscope with nasotracheal tube, acute angle video laryngoscope with oral endotracheal tube	5th attempt	Acute angle video laryngoscope with oral endotracheal tube
Eklund and Voronov, 2017 [23]	4 years	Female	Mandible reconstruction	Not reported	Oral fiberoptic intubation with laryngeal mask airway, oral endotracheal tube, nasal endotracheal tube	Several attempts	Nasal endotracheal tube
Shankar et al., 2022 [24]	6 years	Male	Brain abscess drainage with an external ventricular drain	Ketamine	Laryngoscope with uncuffed oral endotracheal tube	2nd attempt	Laryngoscope with uncuffed oral endotracheal tube

Airway Techniques

As seen in Table 2, when analyzing the initial airway management technique, video laryngoscopy using a curved Macintosh blade with endotracheal intubation (ETT) was the most frequently employed initial strategy as reported in 12 cases (42.9%) [9,13,15,19]. Following this, initial management using video laryngoscopy with a straight Miller blade and ETT was seen in seven cases (25%) [9,14,18,21]. Two cases (7.1%) reported using laryngoscopy during initial intubation attempt, but did not report what type of blade was used [22,24]. LMAs and fiberoptic bronchoscopes were less frequently used as initial intubation techniques, with each being used in two cases (7.1%) [12,16,20,23]. Finally, I-gel, hyperangulated laryngoscopes, and acute angle laryngoscopes were used during first intubation attempts in one case each (3.6%) [10,11,17]. Successful intubation in these patients was seen in 14 cases (50%) [9,11,12,15-17,20], with the remaining cases requiring either multiple attempts as reported in 11 cases (39.3%) [9,13,18,19,21-24], or having a complete failed intubation as reported in three cases (10.7%) [9,10].

Intubation Success Rates

The cases were subsequently stratified based on whether the first attempt at intubation was successful. As seen in Table 3, the first-attempt success had a total of 14 cases, with the most common airway technique being video laryngoscopy and ETT with a curved Macintosh blade, as reported in seven cases (50%) [9,15]. Following this were video laryngoscopes with a straight Miller blade and ETT [9,14] and fiberoptic bronchoscopes [16,20] as seen in two cases, respectively (14.3%). I-gel, LMA, and hyperangulated bronchoscopes were less frequently employed techniques [11,12,17]. Additionally, mask ventilation difficulty was analyzed and showed that six cases (42.9%) had no difficulty with ventilation [12,14-17,20], while the remaining eight cases (57.1%) did not report any findings.

**Table 3:** Successful first intubation cases.

Article (author, year)	Type of anesthesia	Airway technique	Mask ventilation difficulty	Intubation difficulty	Complications
Choudhury and Kapoor, 2017 [9]	Isoflurane	Straight laryngoscope and endotracheal tube	Not reported	None	Delayed extubation
Choudhury and Kapoor, 2017 [9]	Sevoflurane	Curved laryngoscope and endotracheal tube	Not reported	None	None
Choudhury and Kapoor, 2017 [9]	Sevoflurane	Curved laryngoscope and endotracheal tube	Not reported	None	None
Choudhury and Kapoor, 2017 [9]	Ketamine	Curved laryngoscope and endotracheal tube	Not reported	None	Hemodynamic instability
Choudhury and Kapoor, 2017 [9]	Sevoflurane	Curved laryngoscope and endotracheal tube	Not reported	None	None
Choudhury and Kapoor, 2017 [9]	Ketamine	Curved laryngoscope and endotracheal tube	Not reported	None	None
Choudhury and Kapoor, 2017 [9]	Sevoflurane	Curved laryngoscope and endotracheal tube	Not reported	None	Delayed extubation
Singh et al., 2022 [11]	Sevoflurane	I-gel supraglottic airway device	Not reported	None	None
Khan et al., 2017 [12]	Sevoflurane	Laryngeal mask airway	No difficulty	None	None
Torres Salazar et al., 2025 [14]	Sevoflurane	Straight video laryngoscope and cuffed endotracheal tube	No difficulty	None	None
Vitković and Milić, 2016 [15]	Sevoflurane	Curved video laryngoscope and endotracheal tube	No difficulty	None	None
Kim et al., 2020 [16]	Propofol	Transnasal fiberoptic bronchoscope with endotracheal tube	No difficulty	None	None
Molins et al., 2016 [17]	Sevoflurane	Hyperangulated video laryngoscope with cuffed endotracheal tube	No difficulty	None	None
Lokhande et al., 2016 [20]	Propofol	Fiberoptic bronchoscope with cuffed endotracheal tube	No difficulty	None	None

Fourteen cases had an unsuccessful first attempt at intubation and were then further stratified based on the original intubation technique attempted, as seen in Tables 4, 5. Table 4 details five cases (35.7%) that attempted to use a curved Macintosh video laryngoscope with ETT and did not succeed on the first attempt at intubation [9,13,19]. Notably, three of these cases had failed attempts and required other forms of airway management such as tracheostomy and retrograde intubation [9]. Additionally, Table 5 showcases the five cases (35.7%) that attempted to use a straight Miller video laryngoscope with ETT [9,18,21]. Within this group, four of those cases were able to successfully intubate using the straight blade and video laryngoscope after a few attempts [9,18]. One patient who underwent successful straight Miller video laryngoscopy after multiple attempts notably died post-operatively [9]. Other difficult intubation methods were less frequently utilized and had variable success, including one right-angle laryngoscope, one fiberoptic intubation, and two video laryngoscope intubations without blade classification [10,22-24].

**Table 4:** Unsuccessful first attempt at intubation with a curved laryngoscope.

Article (author, year)	Type of anesthesia	Airway technique	Mask ventilation difficulty	Intubation difficulty	Complications	Successful airway strategy
Choudhury and Kapoor, 2017 [9]	Sevoflurane	Curved laryngoscope and endotracheal tube, straight laryngoscope and endotracheal tube, tracheostomy	Not reported	Failed intubation	Ventilator-associated pneumonia	Tracheostomy
Choudhury and Kapoor, 2017 [9]	Sevoflurane	Curved laryngoscope and endotracheal tube, straight laryngoscope and endotracheal tube, tracheostomy	Not reported	Failed intubation	Pulmonary artery crisis	Tracheostomy
Choudhury and Kapoor, 2017 [9]	Sevoflurane	Curved laryngoscope and endotracheal tube	Not reported	Retrograde intubation	None	Retrograde intubation
Sun et al., 2017 [13]	Sevoflurane	Curved video laryngoscope, transnasal fiberoptic bronchoscope with endotracheal tube, transoral fiberoptic bronchoscope	No difficulty	4th attempt	None	Transoral fiberoptic bronchoscope
Ishio et al., 2016 [19]	Propofol	Curved video laryngoscope with endotracheal tube, curved video laryngoscope with spiral endotracheal tube	Not reported	3rd attempt	None	Curved video laryngoscope with spiral endotracheal tube

**Table 5:** Unsuccessful first attempt at intubation with a straight laryngoscope.

Article (author, year)	Type of anesthesia	Airway technique	Mask ventilation difficulty	Intubation difficulty	Complications	Successful airway strategy
Choudhury and Kapoor, 2017 [9]	Sevoflurane	Straight laryngoscope and endotracheal tube	Not reported	5th attempt	Accidental extubation, death	Straight laryngoscope and endotracheal tube
Choudhury and Kapoor, 2017 [9]	Sevoflurane	Straight laryngoscope and endotracheal tube	Not reported	2nd attempt	None	Straight laryngoscope and endotracheal tube
Choudhury and Kapoor, 2017 [9]	Sevoflurane	Straight laryngoscope and endotracheal tube	Not reported	3rd attempt	None	Straight laryngoscope and endotracheal tube
Shukeri et al., 2016 [18]	Not Reported	Straight video laryngoscope, curved video laryngoscope, straight video laryngoscope with endotracheal tube	No difficulty	4th attempt	None	Straight video laryngoscope with endotracheal tube
Munasinghe et al., 2021 [21]	Sevoflurane	Straight laryngoscope with endotracheal tube, laryngeal mask airway	Difficult, oropharyngeal airway used	3rd attempt	None	Laryngeal mask airway

Discussion

Key Findings and Airway Success

This systematic review analyzes 28 cases of anesthetic and airway management in pediatric patients with Goldenhar syndrome. Within these reports, there was a high proportion of cardiovascular and oromaxillofacial procedures, consistent with the multisystem involvement of Goldenhar syndrome. General anesthesia was used in these cases, with sevoflurane being the most frequently employed anesthetic, especially in the younger population [9,11-15,17,21]. This is consistent with current pediatric anesthesia practices, as sevoflurane is preferred due to its smooth induction and rapid emergence. Notably, no clear relationship was analyzed between anesthetic agent and airway management success. This suggests that airway management outcomes are influenced by anatomical factors rather than anesthetic technique.

Airway management strategies varied widely, with video laryngoscopes emerging as the most commonly selected initial approach. Curved Macintosh-style video laryngoscope blades were used most frequently [9,13,15,17], followed by straight Miller-style blades [9,14,18,21]. This pattern reflects the increasing use of video laryngoscopy for anticipated difficult airways. Despite the enhanced glottic visualization, however, successful first-attempt intubation was reported in only half of the cases, emphasizing the complexity of airway management in these patients. When analyzing cases based on first-attempt intubation success, it was found that video laryngoscopy with a curved Macintosh blade was most frequently effective [9,15], followed by a straight Miller blade [9,14]. Fiberoptic bronchoscopy was also reported to be successful in first-attempt intubation; however, this technique was less frequently employed [16,20]. For the cases that were unsuccessful, intubation required multiple attempts and escalation to alternative airway strategies.

Although video laryngoscopes were most commonly employed while undergoing first-attempt intubation, several cases were unsuccessful. Cases that used the curved Macintosh video laryngoscope and did not yield successful intubation on the first attempt ultimately necessitated invasive rescue interventions such as retrograde intubation and tracheostomy [9]. While several cases that attempted straight Miller blade video laryngoscopy were unsuccessful on the first attempt at intubation, cases using this technique saw eventual success after repeated attempts, indicating that blade geometry and improved epiglottic control may contribute to successful intubation in select patients.

Complications

Consistent with the literature [3,4], complications were found to be more frequently reported among patients requiring multiple intubation attempts or rescue techniques. These complications included delayed extubation, respiratory complications, hemodynamic instability, and perioperative mortality [9,24]. Although definitive airway control was ultimately achieved in most patients, these findings emphasize the potential severity of airway-related complications in patients with Goldenhar syndrome and reinstate the importance of minimizing repeated intubation attempts when possible.

Limitations and Future Directions

Limitations of this study are largely due to the small sample size. The available literature on Goldenhar syndrome is primarily case reports, allowing selection and reporting bias to have some influence over data analysis. Study quality was assessed using the NOS. Overall, the included studies were predominantly of low to moderate quality, reflecting the reliance on case reports and small case series within the available literature. Additionally, heterogeneity in clinical reporting may limit interpretability as the intubation techniques, mask ventilation success, and complications were not uniformly and systemically reported between all cases. Cases with more detailed reports could have added more insight into the trends reported.

Despite these limitations, these findings emphasize the need for comprehensive preoperative airway assessment and tailored airway planning in patients with Goldenhar syndrome. Anesthesiologists should anticipate airway difficulty, even in the presence of advanced airway devices such as video laryngoscopy. Immediate availability of alternative airway devices and multiple airway management options, like fiberoptic and surgical airway techniques, should be easily accessible due to the wide variety of airway presentations. Early involvement of advanced airway specialists and predefined rescue plans may reduce airway-related complications and perioperative morbidity and mortality.

## Conclusions

Airway management in patients with Goldenhar syndrome has proven to be challenging and unpredictable, even with the use of advanced airway devices. In this systematic review, patients with Goldenhar syndrome most frequently had video laryngoscopy selected as the initial intubation strategy. Successful first-attempt intubation was found in only half of the cases, with a substantial number of patients requiring multiple attempts or escalation to advanced airway techniques. Additionally, patients who did not achieve successful first-attempt intubation experienced higher rates of perioperative complications.

These findings highlight the critical importance of a comprehensive airway assessment along with individualized airway planning in patients with Goldenhar syndrome. Furthermore, clinicians should anticipate potential airway difficulty and ensure immediate availability of a range of airway management options, including advanced and rescue techniques. Further studies are required to better define the optimal airway management in this high-risk population, as meticulous preparation and multidisciplinary airway expertise remain vital to minimizing airway-related morbidity and mortality in this group.
